# Hyperspectral imaging solutions for brain tissue metabolic and hemodynamic monitoring: past, current and future developments

**DOI:** 10.1088/2040-8986/aab3a6

**Published:** 2018-03-22

**Authors:** Luca Giannoni, Frédéric Lange, Ilias Tachtsidis

**Affiliations:** joptaab3a61 Department of Medical Physics and Biomedical Engineering, University College London, London WC1E 6BT, United Kingdom; l.giannoni@ucl.ac.uk

**Keywords:** biomedical optics, hyperspectral imaging, brain metabolism, brain hemodynamics

## Abstract

Hyperspectral imaging (HSI) technologies have been used extensively in medical research, targeting various biological phenomena and multiple tissue types. Their high spectral resolution over a wide range of wavelengths enables acquisition of spatial information corresponding to different light-interacting biological compounds. This review focuses on the application of HSI to monitor brain tissue metabolism and hemodynamics in life sciences. Different approaches involving HSI have been investigated to assess and quantify cerebral activity, mainly focusing on: (1) mapping tissue oxygen delivery through measurement of changes in oxygenated (HbO_2_) and deoxygenated (HHb) hemoglobin; and (2) the assessment of the cerebral metabolic rate of oxygen (CMRO_2_) to estimate oxygen consumption by brain tissue. Finally, we introduce future perspectives of HSI of brain metabolism, including its potential use for imaging optical signals from molecules directly involved in cellular energy production. HSI solutions can provide remarkable insight in understanding cerebral tissue metabolism and oxygenation, aiding investigation on brain tissue physiological processes.

## 1. Introduction

In recent years, hyperspectral imaging (HSI) has emerged as a promising optical technology for biomedical applications, primarily for life sciences research, but also aimed at non-invasive diagnosis and image-guided surgery [1–4]. It is capable of providing real-time quantitative information for several biological processes in both healthy and diseased tissues. In particular, this is achieved by measuring the intensity changes of numerous different wavelengths of reflected, emitted or fluorescent light after interacting with tissue. These light intensity changes occur due to variations in the optical properties (mainly scattering and absorption) of the target tissue, that can be accounted for both modifications in the biological structure of its components and changes in the concentrations of intrinsic light-absorbing or light-emitting chromophores [5, 6].

HSI systems have then been used to investigate and detect multiple biological phenomena in healthy tissue and in various diseases, such as quantification of blood oxygenation and perfusion [7–10], cancer type differentiation and cancer tissue metabolism [11–13], retinal diseases [14–16], cardiovascular conditions [17–19] and haemorrhagic shock [20, 21]. Different types of tissues have also been targeted using HSI setups, including skin [22–24], oral and gastrointestinal tissue [25–27], breast [28, 29] and brain [30–33].

The present review article aims specifically to summarize the literature regarding the application of HSI for monitoring and investigating brain tissue hemodynamics and metabolism, with major emphasis on the latter phenomenon. This encompasses non-pathological cerebral tissue during both regular metabolic activity (e.g. functional activation and response to different stimuli) and brain metabolism under abnormal conditions, such as tissue hypoxia, hyperoxia and ischemia. The study of cerebral tissue metabolism and hemodynamics is important for developing a deeper and broader understanding of brain tissue physiology, as well as to accurately investigate and map cerebral activity following neuronal activation [34, 35]. Furthermore, monitoring brain metabolism in certain pathological conditions, such as neurodegenerative diseases and brain injuries, potentially allows identification of irregular tissue functionality [36–38]. The possibilities of future potential hyperspectral approaches explicitly for brain metabolic imaging based on established optical solutions will be also discussed.

A brief overview on the application and use of light in brain tissue imaging, with its advantages and limitations, will be provided first. Then, the different definitions of HSI frequently used in the literature will be discussed, with the intent of precisely stating which concept of hyperspectral is applied to the main topic of this article. A general summary of the different components and specific instrumentation used in HSI solutions for monitoring brain metabolism and hemodynamics will follow, together with an outline of the major hyperspectral acquisition techniques. At the end of the current section, an introduction to the main biological mechanisms of brain tissue metabolism and the related optical measurable signals will be then presented.

Afterwards, the article will present HSI for monitoring oxygen delivery and hemodynamics in brain tissue. It is important to clarify that, although a relation between oxygenation of brain tissue and its metabolic activation has been extensively demonstrated [34, 35, 39–41], measuring spatially localized variations in the concentration of oxy-hemoglobin (HbO_2_) and deoxy-hemoglobin (HHb) does not directly quantify brain metabolism [42, 43]. Therefore, specific methods have been proposed and explored in order to also quantitatively monitor metabolic activity in cerebral tissue. The core part of this review will concentrate on their current and future applications with HSI. It will focus principally on two main aspects of brain metabolic monitoring through HSI that includes: (1) quantification of brain oxygen metabolism, which involves imaging cerebral hemodynamics and estimating the cerebral metabolic rate of oxygen (CMRO_2_); and (2) brain tissue energetics, where energy production inside cerebral cells is quantified by imaging specific light-interacting molecules involved in cellular aerobic respiration and in adenosine triphosphate (ATP) synthesis.

### 1.1. The use of light to image and monitor the brain

*In vivo* optical brain imaging modalities, such as HSI, exploit the interaction of light and biological tissue at diverse wavelengths in the electromagnetic spectrum, with the purpose of retrieving physiological information. This has many advantages, the most important are the excellent sensitivity to functional changes and the specificity of particular optical signatures to fundamental molecules and substances in the body, generating what is commonly known as *intrinsic contrast* [31]. As mentioned previously, this contrast depends on the different interactions between light and tissue. Light absorption and scattering are the two chief phenomena occurring when light travels through living matter, such as the brain. Absorption by specific chromophores, particularly water and hemoglobin, limits depth penetration of light into cerebral tissue in the visible range (400–700 nm) to only few mm. However, brain cortex (as any biological tissue in broad sense) is relatively transparent to light in the near-infrared (NIR) range, between 650 and 1350 nm (known as *optical window*), allowing its use for non-invasive mapping of brain hemodynamics and functional activity [1, 5, 6]. Nonetheless, there are critical restrictions to image quality when optical imaging is performed non-invasively, due to the influence of scattering from different layers of the head (primarily skull and scalp). In the NIR range, the brain has high scattering properties due to its structural inhomogeneity [5]. Thus, light diffuses in cerebral tissue, undergoing multiple scattering events in short distances. This causes important limitations to the spatial resolution achievable by any optical imaging systems, since an ideal point illumination will not produce a corresponding point image but a more blurred area. Such an imaging response is called a *tissue point-spread function* and the blurring effect becomes more significant for deeper propagation distances [44]. Therefore, an intrinsic trade-off exists between spatial resolution and depth of penetration for non-invasive optical imaging of the brain.

Animal studies involving the thinning of the skull or even the complete uncovering of the cortex offer direct acquisition of high-resolution images of the surface of the living brain with minimal effects on its normal functions and physiological state. The elimination of the scalp and the skull, with skin layers and vasculature, excludes contamination of the optical signal from sources other than the illuminated brain tissue, as well as greatly enhances both light penetration in the cortex and spatial resolution of the images. Additionally, the smaller the size of the brain of the targeted animals is, the greater the detrimental effect of light scattering in tissue is mitigated, thus gradually improving sensitivity and quantitation power of the optical data. The exposed cortex is thus the preferred imaging target for the HSI studies reported in this article. In particular, the uncovering of the cerebral cortex allows the use of spectral bands in the visible range for imaging, permitting a higher contrast due to the greater absorption in this portion of the electromagnetic spectrum from major chromophores in the brain, primarily the two forms of hemoglobin (as explained in section 2).

Finally, it is important to mention also the role of optical modeling, which is usually implemented to numerically simulate light transport and diffusion in biological tissue and thus to greatly improve image reconstruction and quantification, as well as to estimate optical pathlengths. Monte Carlo stochastic approaches are among the most used and are able to generate very accurate modeling of light propagation inside general-shaped domains and multi-layered media, with the advantage of providing also specific information on individual photon tracks and histories [6, 45].

### 1.2. Definition of HSI for brain metabolism and hemodynamics

HSI has been commonly defined in a broad and general sense as the acquisition of two-dimensional (2D) images across a wide range of the electromagnetic spectrum [1–3, 46, 47]. This definition has sometimes corresponded to a similarly parallel concept in single-point optical spectroscopy, known as broadband spectroscopy [48]. The precise and exact number of wavelengths to be used in HSI has never been clearly established and it frequently depends on the application, stretching from only two wavelengths up to a few hundred. For this reason, the boundary dividing HSI and another similar technique, i.e. multispectral imaging (MSI), is not always distinctly delineated and these two optical imaging approaches can occasionally overlap in the literature, being often subject to arbitrary interpretations. For example, Hillman [31] simply defined HSI as the measurement of two or more wavelengths. On the contrary, other authors [49–53] stated that tens to hundreds of contiguous spectral bands must be involved to create a typical hyperspectral data cube, also known as *hypercube* (i.e. a three-dimensional dataset containing the spatial coordinates of each pixel along the *x* and *y* axes, plus the spectral information along the *z* axis).

**Figure 1.**
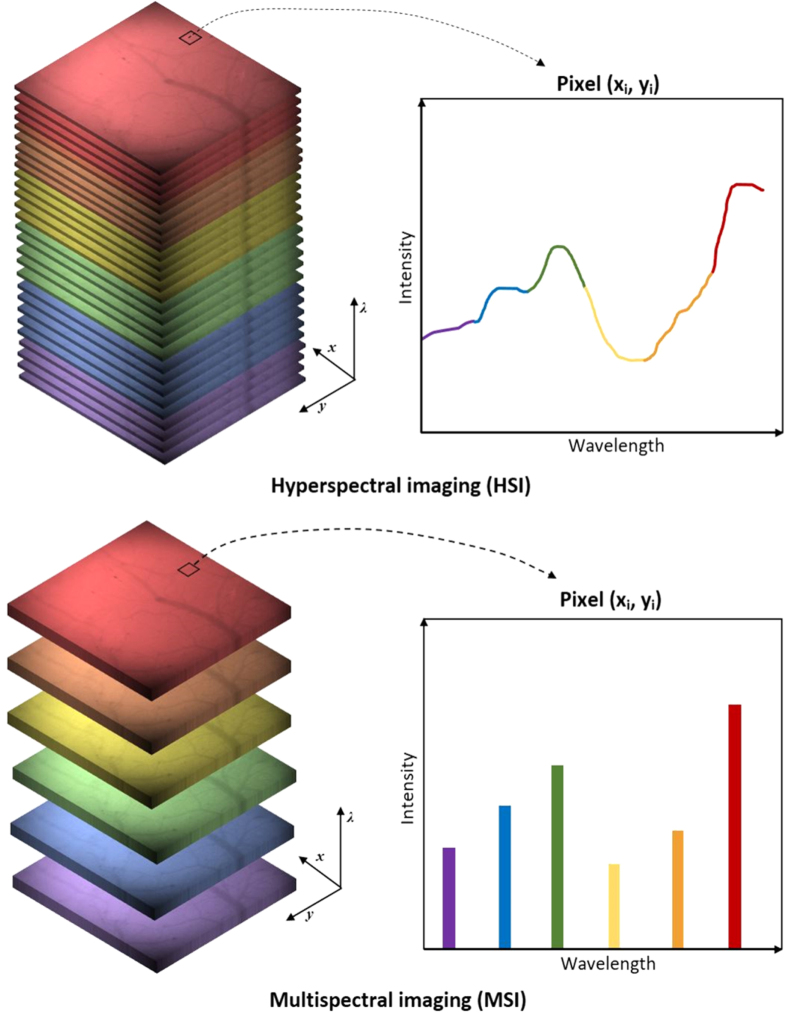
Difference between hyperspectral (top) and multispectral (bottom) imaging. The top image shows the creation of the hypercube using a large number of contiguous spectral bands. The result gives a complete spectrum for each pixel (*x_i_*, *y_i_*). In the bottom image, only discrete and discontinuous portions of the spectral range are considered.

Spectral sampling and resolution are generally considered the key factors that distinguish HSI and MSI (figure 1): while MSI focuses on discrete and relatively spaced wavelength bands, HSI primarily utilizes very narrow and adjacent spectral bands over a continuous spectral range, as to reconstruct the spectrum of each pixel in the image [1, 4, 20, 53]. Again, the width of these bands and their mutual separation are not specifically defined, though they are typically below 10–20 nm [47].

The spectral range covered by a HSI system and the number of wavelength bands it utilizes usually depends on its specific mode of acquisition of the hypercube data. These modes will be discussed in the next paragraph. A larger number of wavelength bands with high spectral resolution and sampling typically requires a more complex HSI instrumentation and it is associated with a larger amount of data to be collected, stored and processed.

For the present review, a hyperspectral system and setup is considered to be any imaging system that involves the use and detection of three or more contiguous wavelength bands, thus slightly extending the definition of Hillman [31]. However, spectral resolution will be still taken into account as a key aspect. The reason behind this choice is related to the narrowness of the spectral ranges typically analyzed for retrieving quantitative information from specific chromophores and molecules involved in metabolic activity *in vivo*. Nonetheless, high spectral sampling and resolution typical of HSI technologies are still strongly required to identify these compounds and to adequately quantify and localize them. Optimal selection of the spectral range and of the specific wavelength bands is also a crucial point in the application of HSI to brain imaging. As stated previously, the visible range is usually the preferred choice for targeting the exposed cortex, while the NIR range can provide a solution for non-invasive and diagnostic approaches. In many cases, optimization of the choice of the spectral bands leads to the use of a relatively small number of wavelengths (typically between six and ten) corresponding to precise portions of the absorption or fluorescence spectra of the targeted chromophores. This has the advantage of reducing the complexity of the involved hyperspectral instrumentation and also the sheer size of the hyperspectral data to handle.

### 1.3. HSI techniques and instrumentation for brain tissue monitoring

The spectral and spatial information that constitutes the hypercube can be collected in different ways, according to the configuration of the HSI system and its instrumentation. The three main hyperspectral image acquisition modes can be summarized as: (1) spectral scanning modes, (2) spatial scanning modes and (3) snapshot modes, also called snapshot imaging spectrometry [1, 49, 54].

Spectral scanning modes consist of the sequential acquisition of 2D images of the target at each required wavelength band. Every acquired image corresponds essentially to a single spatial slice (*x*, *y*) of the hypercube. At each image acquisition, the hypercube is reconstructed by piling up all the slices along the spectral dimension (*λ*). Spectral scanning acquisition can be implemented in various ways, primarily by either filtering the light emitted by the illumination source (excitation-side) or filtering the reflected/emitted light before detection (emission-side). For this purpose, different filtering technologies can be used. Among these, mechanical filtering via filter wheels is one of the most widely employed [1, 4, 31]. It is advantageous for its low complexity and cost, though the number of spectral bands that can be installed in the wheel is usually limited (up to 8–10), as well as the spectral resolution and the selection of the specific wavelengths required. In addition, the switching speed between filters is typically slow [1]. Tunable filters, such as acousto-optical tunable filters (AOTFs) and liquid crystal tunable filters (LCTFs), can provide much higher image quality and better spectral resolution and sampling over a wide range of wavelengths. They also offer rapid tuning rates that can significantly reduce the total acquisition time for a single hypercube [1, 49]. As an alternative to filtering, spectral scanning can also be performed by directly switching between multiple sources with narrow spectral emission, such as light-emitting diodes.

Spatial scanning modes acquire spatio-spectral information at each scan of succeeding portions of the 2D image. They can collect the complete spectrum (*λ*) of each pixel of the image, point by point, and then reconstruct the entire hypercube at every spatial location (*x*, *y*). This approach is commonly called point scanning or *whiskbroom* scanning. Alternatively, the spectral data (*x*, *λ*) of a single array of pixels can be acquired at each scan to build the hyperspectral dataset line by line along the other spatial dimension (*y*). In this case, the acquisition mode is known as linear scanning or *pushbroom* scanning. Both of the two modalities require a relative movement between the subject and the hyperspectral setup and, compared to spectral scanning, do not provide a real-time display of the whole field of view (FOV) of the image. Collimating devices and optical spectrometers are typically necessary to accomplish the spatial scanning procedure: pinhole apertures (for point scanning) or slits (linear scanning) collimate light to image (or illuminate) only a portion of the FOV; while dispersive devices, such as diffraction gratings and prisms, are used to separate the polychromatic light into its constituent wavelengths [1].

Snapshot HSI techniques encompass a number of acquisition approaches that normally share the capability of recording every image at each spectral band simultaneously, within a single integration time of the detector. These modes totally eliminate the need of scanning procedures. Most snapshot techniques used for brain tissue metabolic monitoring rely on dispersive elements, like prism arrays and mirrors, in order to split the FOV of the imaged subject into sections (slices or portions of the image) that are then separated in their spectral constituents. After that, the sections are distributed and recorded over the whole detecting area. By doing that, the detector is divided in regions that embed the complete spatial and spectral information of the target, from which the hypercube has to be reconstructed. These types of snapshot modalities are usually called *image mapping spectrometers* (IMS) [49, 55]. The simplest configuration involves multiplexing the detector area of the full image at each wavelength. Alternatively, *image slicing spectrometers* separate the image in slices. However, IMS approaches are generally limited by the dimension of the detector area, which consequently restricts the size of the hypercube and the spatial resolution of the images [49, 55].

Generally, HSI acquisition via spectral or spatial scanning provides higher spectral resolution and enables the use of a greater number of wavelengths bands, although at the cost of a longer imaging time. They are also more sensitive to motion artifacts and distortions in the final reconstructed image, which may occur if the subject moves during the time of a single hypercube acquisition. This may produce significant issues especially for *in vivo* applications [1, 4, 54]. Contrariwise, snapshot HSI systems have the advantage of reducing the acquisition time of each hyperspectral dataset, since they can capture an entire hypercube in just a single detector integration period, without the need for filtering or relative movement between the subject and the HSI system. Nonetheless, this is typically obtained at the expenses of both spectral sampling and spatial resolution, due to the very large volume of data handled [1, 4, 55].

In almost every application of HSI in imaging of brain metabolism and hemodynamics, illumination of the target is normally delivered by white light sources encompassing a broad range of wavelengths, e.g. halogen lamps or gas discharge lamps. Laser sources are not common, although they may be used in HSI setups for complementary imaging techniques, such as for laser speckle contrast imaging (see section 3). Still, an alternative way to provide broadband illumination is represented by supercontinuum lasers, especially in spectral acquisition mode, due to their large spectral range (typically covering visible and infrared emission), high intensity and power output, as well as for their fast repetition rates. However, they are typically more expensive compared to conventional light sources.

For light intensity detection, charge-coupled devices (CCDs) are the most extensively used sensor instruments for hyperspectral measurements of brain metabolism and hemodynamics in any acquisition mode, due to their high quantum efficiency and low dark noise [1]. They represent a well-established detection technology at relatively moderate cost and can cover large spectral ranges, spanning from visible to NIR light. Nonetheless, complementary metal-oxide sensors (CMOS), particularly the so-called scientific CMOS (or sCMOS), are rapidly emerging as alternative imaging detectors for the light intensities involved in HSI, as they can combine fast frame rates with high dynamic ranges. Commercial hyperspectral cameras, mostly based on snapshot mode, have also started to appear on the market in recent years and have been used for brain tissue metabolic and hemodynamic imaging. Spatial resolution of the reviewed HSI systems varies from recording only spread and featureless 2D responses in the brain (in the order of cm or mm) to visualization of small details of the cerebral vasculature (down to a few *μ*m), mostly depending on the type of detector, the FOV, the used reconstruction algorithm, as well as the selection of the spectral bands.

Table 1 provides an overview of the representative HSI systems that will be discussed in the next sections for imaging and monitoring metabolic and hemodynamic activity of brain tissue. Their characteristics are summarized, together with their instrumentation, the image acquisition mode, the metabolic parameters that they target and the triggering stimulus or condition.

**Table 1.** Features, instrumentation and acquisition methods of every representative HSI system reported in the current article for brain metabolic and hemodynamic monitoring.

References	Spectral range (nm)	Number of spectral bands	Spectral resolution (nm)	Acquisition mode	Illumination setup	Detection setup	Metabolism target	Targeted stimulus or condition
Malonek [87]	500–700	—	1–4	Linear scanning	White light source	CCD + slit + diffraction grating	Tissue oxygenation	Visual stimulation
Devor [89]	560–610	6	10	Spectral scanning	White light source + filter wheel	CCD	Tissue oxygenation	Whisker stimulation
Konecky [91]	484–652	38	3–8	Snapshot	White light source	CCD + prism array	Tissue oxygenation	Whisker stimulation
Shonat [94]	504–600	12	5	Spectral scanning	White light source	CCD + AOTF	Tissue oxygenation	Hypoxia and hyperoxia
Pichette [98]	481–632	16	∼15	Snapshot	White light source	Commercial snapshot camera	Tissue oxygenation	Epileptic spikes
Dunn [88], [89], [109]	560–610, 768	7	10	Spectral scanning	White light source + filter wheel + laser	CCD	CMRO_2_	Tactile stimulation
Jones [110]	560–610, 768	7	10	Spectral scanning	White light source + filter wheel + laser	Two CCDs	CMRO_2_	Focal cerebral ischemia
Gao [134]	∼480–600	25	5.6	Snapshot	White light source + filters	CCD + image slicer	Fluorescence	Fluorescent phantom model
Yin [95]	450–650	9	20	Spectral scanning	White light source	CCD + LCTF	CCO	CSD

**Note**. CCD, charge-coupled device; AOTF, acousto-optical tunable filter; CMRO_2_, cerebral metabolic rate of oxygen; LCTF, liquid crystal tunable filter; CCO, cytochrome-c-oxidase; CSD, cortical spreading depression.

### 1.4. Measuring metabolic brain activity and hemodynamics

Functional metabolic activity in the brain can be generally defined as the sum of all the biochemical processes associated with regular cerebral functions, including those that keep brain cells alive and enable them to execute their biological roles in the tissue. Even at rest, the metabolic energy demand of the brain is considerable, being one of the highest energy-consuming organs. It utilizes about 20%–23% of the total energy requirement of the human adult body, despite representing only 2%–2.3% of its overall weight [56]. When the brain is subject to metabolic activation due to signaling events stimulating cerebral cells, an overall increase in ATP demand normally occurs. This energy demand is higher than the typical ATP demand in the basal resting state, which is set by the specific experimental protocol used in the measurements. Thus, baseline metabolic activity during rest condition usually varies depending on the state of the subject, being for instance either awake, unconscious, anesthetized or even comatose [34].

The detection and measurement of cerebral metabolic signals through imaging enables to identify and localize changes in brain activity and function under several conditions: from resting state, through functional activation and response to stimuli, to irregular physiological circumstances, such as during tissue hypoxia, hyperoxia and even acute ischemia. In particular, these abnormal conditions can disrupt normal metabolism in deleterious ways, for instance due to oxygen deficiency (in the cases of hypoxia and ischemia), and subsequently lead to severe damages to cerebral tissues [57].

**Figure 2.**
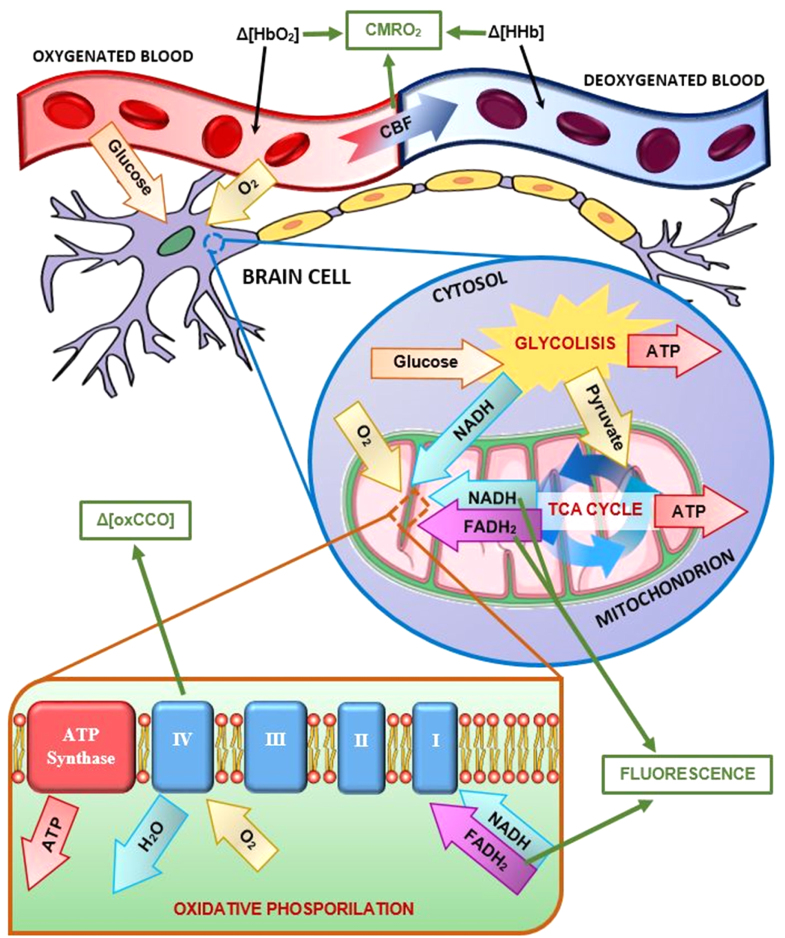
Diagram of the major metabolic pathways in brain tissue. Key metabolites and processes are reported, and related optical signals and parameters (in green squares) can be measured and estimated using different HSI approaches: namely, quantification of CMRO_2_, detection of fluorescence from NADH and FAD, plus monitoring of the redox states of CCO.

Different and interconnected energy-producing pathways compose the overall metabolic activity of brain tissue: among them, the two major ones are *glycolysis* and *oxidative metabolism*, both cardinal in cellular respiration. Parameters related to these processes and optical signals from compounds directly involved in their key reactions, as either metabolites or products, can be measured independently via HSI techniques [34, 56, 58]. Figure 2 summarizes the main metabolic pathways that can be targeted via HSI and the corresponding parameters and optical signals used to quantify brain metabolic activity.

Glycolysis occurs in the cytosol of cells and, in aerobic conditions, it converts glucose (C_6_H_12_O_6_) into pyruvate (CH_3_COCOO^−^). The free energy released by this process is used to produce ATP and reduced nicotinamide adenine dinucleotide (NADH). ATP is well known for being the fundamental molecule involved in the storage and transport of chemical energy within cells to fuel and drive their core functions. NADH is another key coenzyme involved in the oxidative metabolism that generally follows glycolysis, acting as an electron donor in the electron transport chain (ETC). NADH and pyruvate are then transferred inside the mitochondria, where oxidative metabolism takes place [34, 58–60].

During oxidative metabolism, pyruvate is first converted into acetyl-CoA inside the mitochondrial matrix, creating also additional NADH and carbon dioxide (CO_2_) as a waste by-product. Subsequently, acetyl-CoA is oxidized to CO_2_ in the tricarboxylic acid (TCA) cycle, also called *Krebs cycle*, where water is consumed and further ATP and NADH are produced. In the TCA cycle, another important molecule is also generated, called reduced flavin adenine dinucleotide (FADH_2_), which like NADH it is utilized as a redox agent in the final stage of oxidative metabolism, called *oxidative phosphorylation*. Oxidative phosphorylation occurs in the inner membrane of the mitochondria and primarily involves a series of redox reactions ultimately releasing chemical energy used to synthetize additional ATP. These redox reactions are mediated by four protein complexes linked together to form the ETC. NADH and FADH_2_, produced in the early phases, initiate such process by donating electrons and converting into their corresponding oxidized forms, i.e. NAD and FAD, respectively. The electrons are then transferred through each of the ETC complexes. Oxygen (O_2_), carried by hemoglobin in the blood, diffuses into brain cells and receives the transported electrons via complex IV (also known as cytochrome-c-oxidase, CCO) in the ETC, converting O_2_ into water and CO_2_. Finally, the redox potential change in the ETC drives the production of ATP through another large enzyme complex, called ATP synthase [58–60].

It is evident that oxygen plays a crucial role in oxidative metabolism, being the ultimate electron acceptor leading to energy production and thus it is a key component, together with glucose, for fueling the entire metabolic activity of brain tissue. Moreover, the net amount of ATP produced during oxidative phosphorylation, equal to about 30–32 molecules, greatly surpasses that generated via glycolysis and all other metabolic pathways, which total around five units [34]. Hence, mapping oxygen delivery and consumption across regions of the brain is one of the ways to directly measure and quantify brain metabolism. Nonetheless, monitoring just the changes in concentrations of the two states of hemoglobin, i.e. oxygenated (HbO_2_) and deoxygenated (HHb), in a specific area of the brain does not explicitly assess O_2_ consumption in metabolism. It is an index of oxygen demand and delivery to brain tissue during rest conditions and under functional activation [61]. Brain oxygenation and hemodynamics mapping is also fundamental to evaluate brain operation during oxygen-dependent conditions, such as hypoxia, hyperoxia and ischemia [62]. Functional magnetic resonance imaging (fMRI) and functional NIR spectroscopy (fNIRS) are the major modalities that have been used to achieve this purpose [63–65]. In particular, HSI for brain oxygenation has relied on approaches and methodologies similar to those used in fNIRS, which will be largely discussed in section 2.

The principal parameter that is utilized to quantify cerebral oxidative metabolism is CMRO_2_. CMRO_2_ is the rate of oxygen consumption by the brain and it is strongly related to the cerebral blood flow (CBF) [34, 66]. It can be measured directly *in vivo* using positron emission tomography, by imaging the uptake of radiolabelled ^15^O compounds [67]. However, indirect assessments of CMRO_2_ from hemodynamic parameters have also been proposed and investigated via fMRI [68] and fNIRS [69]. Section 3 will describe extensively how HSI has been applied so far to estimate CMRO_2_ from measurements of CBF and other hemodynamic parameters.

Finally, optical imaging techniques have the capability of assessing cellular energetics and ATP production *in vivo* during brain metabolic activity, thanks to the light-dependent properties of some of the molecules that are actively involved in these processes. In particular, both FAD and NADH are autofluorescent in the visible range. Thus, by exciting these compounds in brain tissue with specific wavelengths and measuring the intensity of the emitted fluorescence, it is possible to quantify changes in their redox states, which consequently indicate variations in the rate of production of ATP during metabolism [70–72]. FAD and NADH have also been targeted by HSI systems, taking advantage of their superior spectral resolution and high number of wavelength bands to simultaneously resolve their corresponding fluorescent signals. These approaches can thus provide a new way to assess brain metabolism. This will be discussed in section 4.

Fluorescent exogenous analogs of key molecules involved in cellular metabolic production of energy could also be employed together with HSI techniques: this will also be briefly mentioned in section 4, although without being extensively discussed in this article.

Similarly, the difference in the absorption spectra of the reduced and oxidized states of CCO can be used to measure its *in vivo* concentration. As seen previously, its redox states are associated with O_2_ reduction and consumption in oxidative metabolism. Therefore, monitoring and localizing CCO in cerebral tissue can potentially provide another marker to quantify brain metabolic activity. Studies will be reported in section 4, on the use of broadband fNIRS to measure CCO during brain functional activation and under hypoxic conditions [60, 73]. Section 4 will also cover the feasibility and proposals of applications of HSI to target and image CCO, as a possible future solution for quantifying oxidative metabolism in the brain.

## 2. HSI of brain tissue oxygenation during metabolic activity changes

**Figure 3.**
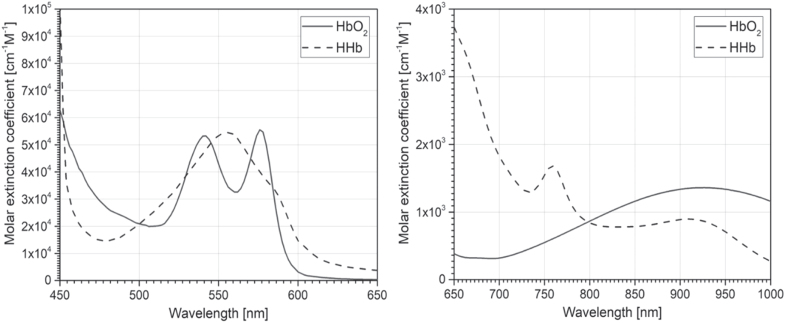
Molar extinction spectra of HbO_2_ (solid) and HHb (dashed) in the visible (left) and NIR range (right). The values are taken from Scott Prahl [74] for the visible range and from UCL Biomedical Optics Research Laboratory (BORL) database [75] for the NIR range.

So far in the literature, the use of HSI to map localized changes in oxygenation of various regions of the brain is the most reported application of this optical technology to cerebral tissue metabolic and hemodynamic monitoring. The *in vivo* measurement of the intrinsic contrast provided by HbO_2_ and HHb has been an established pillar of fNIRS and diffuse optical imaging (DOI) since its first demonstration by Jöbsis in the 1970s [64, 73], due to the distinctive absorption spectra of these two chromophores (see figure 3) and the advantage of the optical window (as mentioned earlier).

The two states of hemoglobin are the major absorbing substances in the brain in the visible and NIR regions. Therefore, changes in their relative concentrations can be resolved by measuring variations in the absorption of brain tissue during oxygenation and perfusion. The use of multiple wavelengths between 400 and 1000 nm permit to obtain superior sensitivity to these changes and better discrimination between HbO_2_ and HHb [7, 49, 52]. For this reason, the higher spectral resolution and narrower bandwidth of HSI technologies are suitable for this task, having the potential to acquire more precise and exhaustive spectral information of the two compounds.

As mentioned before, mapping brain tissue oxygenation does not provide direct quantitative measurements of brain metabolism; nevertheless, it is still a significant indicator of brain metabolic activation. Functional activation in the brain results in local variations of blood flow, blood volume and oxygen delivery and consumption, in order to fuel the increased neural activity [76–78]. A typical cortical response to a stimulus involves an increase in the local concentration of HbO_2_, due to increased oxygen supply, along with a reduction in HHb [77, 78]. The total concentration of hemoglobin (HbT), given by the sum of the concentrations of HbO_2_ and HHb, also shows an increase, although smaller than that in HbO_2_. The concentration of HbT can be derived from the sum of the measured concentrations of HbO_2_ and HHb, or via a direct measurement through the selection of wavelength bands corresponding to the isobestic points of the absorption spectra of the two chromophores (at around 500, 529, 570 and 798 nm) [31]. The overall timing of the hemodynamic response generally varies according to different parameters, such as the specific region of the brain cortex involved, the type of triggering stimulus, the physiological conditions of the subject (e.g. blood pressure), as well as the particular state of hemoglobin considered. Nonetheless, the whole reaction has a typical duration in the order of seconds to tens of seconds, with a delay usually between the beginning of the stimulus and the peak of the hemodynamic changes [31, 78].

The hemodynamic response of brain tissue to altered oxygenation conditions, such as hypoxia, hyperoxia and ischemia, is more complicated and heterogeneous than the response to functional activation, due to the occurrence of larger systemic and physiological effects. Examples of these effects are variations in respiratory rate, ventilation and heart rate [79]. The magnitude and timing of the hemodynamic response are also dependent on the magnitude of the reduced (or augmented) oxygenation, reaching maximum severity in cases of acute ischemia and anoxia. In controlled experimental protocols, the amount of oxygenation is normally set by the fraction of inspired oxygen (FiO_2_). Again, it is possible to identify a common and established trend for the changes in concentrations of the two states of hemoglobin.

Typically, transient hypoxia induces a progressive decrease in HbO_2_ and increase in HHb, which become more severe as FiO_2_ is reduced. Both HbO_2_ and HHb eventually tend to plateaus as the hypoxic condition is maintained with time. HbT also usually shows a general rise, due to vascular dilatation and increase in heart rate [60, 79, 80]. In ischemia, which is typically induced by surgically blocking major brain vasculature, the trend is equivalent to that of hypoxia but with larger spatial extent and higher magnitude in the variations of HbO_2_ and HHb, as well as significant drop in HbT [57]. Conversely, a reverse situation can be observed in the case of hyperoxia, where HbO_2_ increases and HHb diminishes with a similar slow tendency to stable new levels. The incremented oxygen supply also results in an increase in HbT due to the larger concentration of oxygen in the blood [81, 82].

The majority of the HSI methodologies used to calculate the changes in the concentrations of HbO_2_ and HHb from measurement of light attenuation (in each pixel of the hyperspectral images) are based on the modified Beer–Lambert law (MBLL), developed by Delpy *et al* [83]. The MBLL correlates the attenuation *A* of light intensity through brain tissue at a specific time *t* and wavelength *λ* with the concentration *c*_*i*_ of a number of *N* targeted chromophores, as following:

1 \begin{eqnarray*}A(t,\lambda )=-{\mathrm{log}}_{10}\left(\displaystyle \frac{I(t,\lambda )}{{I}_{0}(t,\lambda )}\right)\\ \,\,\,=\,\displaystyle \sum _{i=1}^{N}{\varepsilon }_{i}(\lambda ){c}_{i}(t){D}_{a}(\lambda )+G(\lambda ),\end{eqnarray*}

where *I* is the detected intensity, *I*_0_ is the incident intensity in the tissue area, *ε*_*i*_ is the molar extinction coefficient of the *i*th chromophore, *c*_*i*_ is the concentration of the *i*th chromophore and *G* is a geometrical factor assessing scattering-related loss of light intensity in the brain. The differential optical pathlength *D*_*a*_(*λ*) accounts for the wavelength-dependent increment in the distance traveled by the photons in the tissue due to multiple scattering. Therefore, it represents the ‘true’ optical pathlength effectively covered by the light in the cerebral tissue, which is can be also indicated as *PL* [84, 85].

The application of the MBLL to measured intensity spectra does not normally permit to calculate absolute concentrations, but only relative temporal changes Δ*c*_*i*_ in concentrations during a specific time interval Δ*t* between a specific time point *t*_1_ and an initial time *t*_0_, usually corresponding to the basal rest condition. In this way, by looking at the variation in Δ*A* in Δ*t*, it is assumed that the change in the scattering properties of brain tissue is small compared to the change in absorption due to the considered chromophores; it is also assumed that the incident light intensity *I*_0_ is stationary in time. Thus, the terms *G* and *I*_0_ can be canceled out from the derived equation:

2 \begin{eqnarray*}{\rm{\Delta }}A({\rm{\Delta }}t,\lambda )=-{\mathrm{log}}_{10}\left(\displaystyle \frac{I({t}_{1},\lambda )}{I({t}_{0},\lambda )}\right)\\ \,\,\,\,=\,\displaystyle \sum _{i=1}^{N}{\varepsilon }_{i}(\lambda ){\rm{\Delta }}{c}_{i}({\rm{\Delta }}t){D}_{a}(\lambda ).\end{eqnarray*}

Applying the previous equation (2) to the case of HbO_2_ and HHb and extending it to an arbitrary number *M* of wavelength bands *λ*_*n*_ (according to the HSI setup), a system of *M* linear equations can be obtained. The system can be solved for the temporal changes of concentrations Δ[HbO_2_]_*x*,*y*_ and Δ[HHb]_*x*,*y*_ in each pixel (*x*, *y*) of the acquired hyperspectral images, provided that the molar extinction coefficients ${\varepsilon }_{{{\rm{H}}{\rm{b}}{\rm{O}}}_{2},\lambda n}$ and *ε*__HHb,*λn*__ of the two chromophores at each involved wavelength are available:

3 \begin{eqnarray*}\left[\begin{array}{c}{\rm{\Delta }}{[{{\rm{HbO}}}_{2}]}_{x,y}\\ {\rm{\Delta }}{[{\rm{HHb}}]}_{x,y}\end{array}\right]\,=\,{\left[\begin{array}{cc}{\varepsilon }_{{{\rm{HbO}}}_{2},{\lambda }_{1}} &amp; {\varepsilon }_{{\rm{HHb}},{\lambda }_{1}}\\ {\varepsilon }_{{{\rm{HbO}}}_{2},{\lambda }_{2}} &amp; {\varepsilon }_{{\rm{HHb}},{\lambda }_{2}}\\ \vdots &amp; \vdots \\ {\varepsilon }_{{{\rm{HbO}}}_{2},{\lambda }_{M}} &amp; {\varepsilon }_{{\rm{HHb}},{\lambda }_{M}}\end{array}\right]}^{-1}\\ \,\times \,\left[\begin{array}{c}{\rm{\Delta }}A{({\rm{\Delta }}t,{\lambda }_{1})}_{x,y}/{D}_{a}({\lambda }_{1})\\ {\rm{\Delta }}A{({\rm{\Delta }}t,{\lambda }_{2})}_{x,y}/{D}_{a}({\lambda }_{2})\\ \vdots \\ {\rm{\Delta }}A{({\rm{\Delta }}t,{\lambda }_{M})}_{x,y}/{D}_{a}({\lambda }_{M})\end{array}\right].\end{eqnarray*}

Accurate estimates of the differential optical pathlength *D*_*a*_(*λ*_*n*_) at each wavelength are necessary in order to calculate the relative changes in the concentrations of HbO_2_ and HHb. In fNIRS, direct assessments of this optical pathlength can be performed by measuring the time-of-flight of photons in the targeted tissue area [83]: such approach is advantageous for this acquisition configuration, involving the detection of diffused NIR light and relatively large source-to-detector distances (normally few cm). However, for HSI applications that normally measure reflected photons consisting also of visible light, the optical pathlength is generally very small (∼1–2 mm), thus the temporal resolution requirements to measure the time-of-flight are more difficult to achieve. Therefore, indirect estimations of *D*_*a*_(*λ*_*n*_) are largely derived from numerical analysis based on the diffusion theory and on Monte Carlo simulations [85]. Furthermore, HSI requires the 2D distribution of the source illumination on the imaged area to be taken into account for a correct evaluation of the optical pathlengths in reflectance mode imaging.

HSI systems using the MBLL have been mostly used to monitor hemodynamics response in brain tissue during metabolic activation by targeting *in vivo* the exposed cortex of animal subjects, especially rodents and cats [86–91]. Malonek *et al* [87] implemented one of the first applications of linear scanning HSI to functional brain mapping, by targeting the exposed visual cortex of an anesthetized cat with visible light in the range 500–700 nm and spectral resolution spanning between 1 and 4 nm. The HSI system used a slit and a dispersing grating assembly to acquire sequential spatio-spectral images and reflection spectra of brain microcirculation and hemoglobin response to variable visual stimulation. This showed highly localized hemodynamic responses to the stimulus in specific cortical regions within the first 3 s, with concentration changes spreading to larger distances of several millimetres in the later phase of metabolic activation.

**Figure 4.**
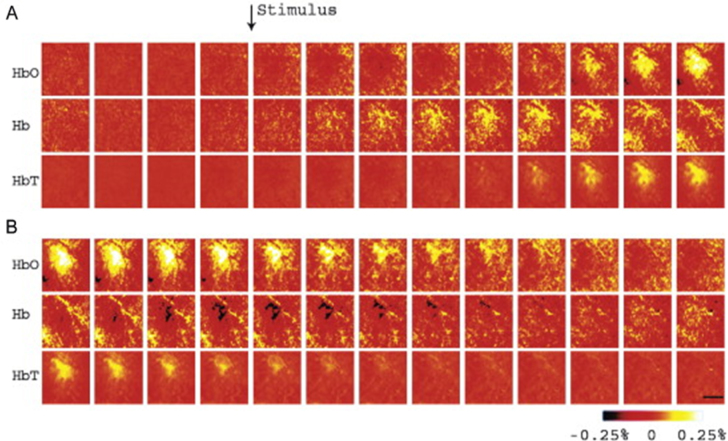
HbO_2_, HHb and HbT spatio-temporal mapping in the exposed somatosensory cortex during tactile stimulation. Each hyperspectral frame was acquired every 200 ms (series (B) is a continuation of (A)), with the black scale bar set at 500 *μ*m. The changes in concentrations are expressed as percentage variations respect to baseline (60 and 40 *μ*M, for HbO_2_ and HHb respectively). Reprinted from [89], Copyright (2003), with permission from Elsevier.

Similarly, Devor *et al* [89] investigated the hemodynamic response of the brain to functional activation, as well as the coupling between neural activity and blood oxygenation during metabolic activity changes. For this purpose, a spectral scanning imaging system was tested on the exposed somatosensory cortex of rats during whisker stimulation. The setup was composed of a mercury xenon arc lamp filtered with a 6-position rotating filter wheel and it employed just six contiguous wavelengths (560, 570, 580, 590, 600 and 610 nm) at a switching rate of about 3 Hz. Each spectral image was then acquired sequentially using a cooled 12-bit CCD camera. Calculations of the variations in HbO_2_ and HHb were performed using the MBLL and expressed in percentage change maps relative to baseline concentrations of 60 and 40 *μ*M, respectively; while changes in HbT were obtained as the sum of the values of the previous two chromophores. The differential optical pathlength at each wavelength was estimated using Monte Carlo simulations based on Kohl *et al* [85]. The calculated oxygenation change maps, showed in figure 4, spatially localized an initial increase in HHb, but also reported an equivalent rapid decrease in HbO_2_ that balanced such effect, leading to a stationary level of HbT during the initial phase after the stimulus. These hemodynamic results were then compared to simultaneous electrophysiological recordings in the same region of the brain using metallic electrodes, which quantified synaptic electrical activity: the comparison between these two different signals indicated a nonlinear and unsynchronized relationship between the neural activation and the hemodynamic response.

A critical factor to be considered in HSI of the exposed cortex is the depth of penetration traveled by the detected light in the brain tissue. Since this reflected light usually has penetrated few millimetres in the cortex, the overall measured signal results to be composed of weighted contributions belonging to various layers of cerebral tissue (i.e. surface of the cortex and parenchyma), where optical properties and vasculature structure differ. Therefore, this generates a difficulty in localizing precisely the depth in the cerebral cortex at which the hemodynamic response occurs. The problem is further complicated by the fact that the penetration depth is also wavelength-dependent, since photons at different wavelengths experience different optical pathlengths in the same brain region.

**Figure 5.**
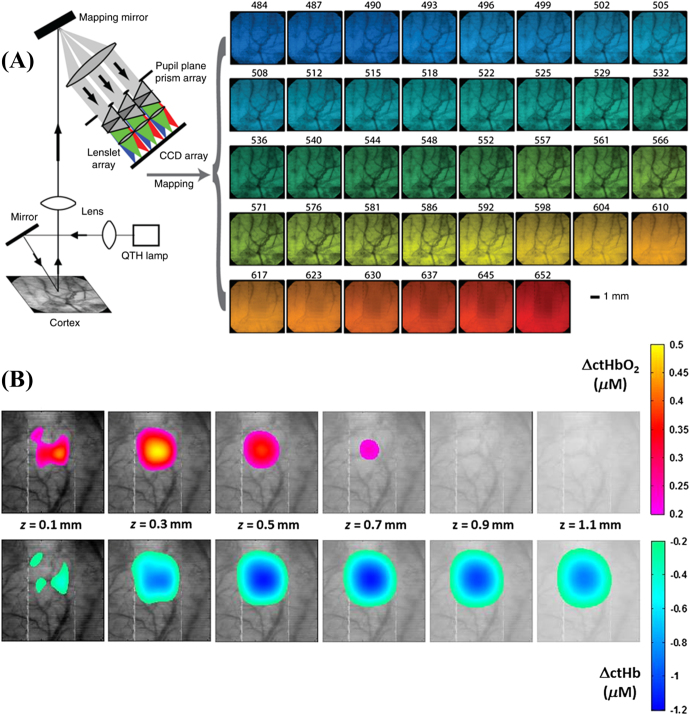
(A) Snapshot HSI setup used to image rat somatosensory expose cortex at 38 spectral bands during a single 50 ms exposure. The 2D images for each wavelength were obtained using mapping reconstruction from dispersed light though a prism array. (B) The changes in concentration of HbO_2_ and HHb were reconstructed using a Rytov tomographic algorithm to generate cross-sections of the hemodynamic response at different depths *z* in brain tissue after 4 s post-stimulus. Reproduced with permission from [91].

A recent work by Konecky *et al* [91] tried to assess the issue of depth sensitivity by using a diffuse optical tomography (DOT) approach to HSI of rat somatosensory cortex under single whisker stimulation. They developed a snapshot HSI system [92] using 38 spectral bands from 484 to 652 nm at an image rate of 5 Hz. The spectral resolution of the system varied from 3 to 8 nm, while the imaged FOV was 6 × 6 mm. In addition to the application of the MBLL to calculate changes in HbO_2_ and HHb, they also implemented the Rytov approximation [93], typical of DOT image reconstruction, analyzing the depth-dependent distortion of the reflectance spectra in order to localize the hemoglobin variations at various depths in the brain tissue. The results of the investigation reported a peak in the hemodynamic response after 3 s post-stimulus using the MBLL, while the Rytov approximation estimated a longer time to peak of about 4 s. The magnitude of the concentration changes calculated with the Rytov tomographic reconstruction were also larger than those obtained by the MBLL, due to the partial volumetric effects the Rytov approximation accounted for. The DOT-based approach was also able to localize the maximum of the hemodynamic response at less than a millimetre beneath the surface of the cortex (precisely, 0.29 ± 0.02 mm for HbO_2_ and 0.66 ± 0.04 mm for HHb). The configuration of the HSI system and part of the results of the study are shown in figure 5. The difference in the depth at which the maximum variations in HbO_2_ and HHb occurred during functional activation may be attributed to a rapid depletion of oxygen in the superficial cortex, followed by a reperfusion of oxygenated blood in the same region. It could also be related to the different distribution of arterioles and venules in brain tissue layers, although the authors also suggested a potential technical limitation of the system to fully resolve the depth of the two chromophores.

The targeting of the exposed cortex of small animals using HSI has also been a preferential choice in several studies assessing hemodynamic response and brain tissue oxygenation during induced hypoxia and hyperoxia [94–97]. Shonat *et al* [94] were among the first to use HSI with AOTFs to study the hemodynamic variations in the exposed cortex of mice under different FiO_2_ levels, from normoxia (FiO_2_ = 21%), through moderate hyperoxia (FiO_2_ = 60%), to hypoxia (FiO_2_ = 10%). The AOTFs permitted a rapid sequential acquisition of 12 wavelengths from 504 to 600 nm (5 nm bandwidth and 8 nm increments) by filtering the reflected light before detection using a slow-scan CCD camera. The reflectance spectra were then used to calculate changes in HbO_2_ and HHb using the MBLL and from these, hemoglobin saturation (SO_2_) maps were derived. The results of these SO_2_ maps were ultimately compared with oxygen tension (PO_2_) maps obtained simultaneously via phosphorescence lifetime imaging: the magnitude of the expected increase in SO_2_ (as tissue oxygenation was varied from hypoxia, through normoxia, to finally hyperoxia) was found to match that of PO_2_, except for the final increment in SO_2_ during hyperoxia. This did not mirror the larger change of PO_2_, possibly indicating that hemoglobin was close to saturation in that condition.

**Figure 6.**
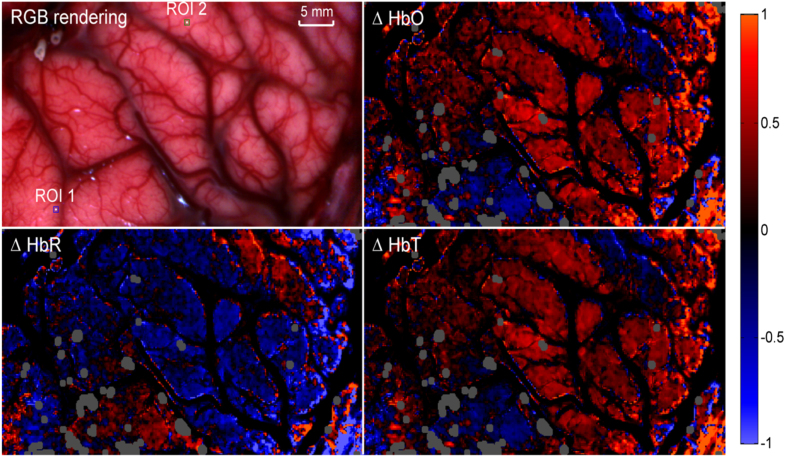
*In vivo* intraoperative HSI of the hemodynamic response in human exposed cortex: the top-left image shows the RGB image of the cortex, while the remaining pictures present the maps of the calculated relative changes in the concentrations of HbO_2_, HHb and HbT (clockwise from top-right). Contrast outside the major vessels (dark areas) was enhanced in order to highlight the small oxygenation changes in the capillary regions. The gray areas correspond to eliminated saturated regions of pixels, due to specular reflection. Reproduced with permission from [98].

HSI imaging of brain tissue hemodynamics during neural activation has not been only confined to small animal imaging. Several attempts to apply hyperspectral approaches to the human exposed cortex are present in the literature, primarily during epilepsy surgery [98–100]. Pichette *et al* [98] recently tested a proof-of-concept snapshot HSI system combining a neurosurgical microscope with a commercial hyperspectral camera for simultaneous detection of 16 spectral bands ranging from 481 to 632 nm, with spectral resolution of about 15 nm (FWHM). This setup was utilized on a fully exposed adult cortex before epileptogenic tissue resection, at a frame rate of 20 fps and acquisition time of 40 ms per frame. The resulting changes in concentrations of HbO_2_, HHb and HbT were calculated using the MBLL, together with a spectral unmixing algorithm, and provided hemodynamic monitoring of the resting metabolic state of human brain (figure 6). Small changes in the hemodynamic response compared to the baseline were attributed to a mix of Mayer waves, vasomotion and epileptic spikes.

Before concluding this section, it must be noted that using MBLL to map hemodynamic changes during brain metabolic activation is not the only approach to hyperspectral data processing that has been investigated. Multivariate analysis algorithms, such as principal component analysis [101, 102] and independent component analysis [103], have also been implemented to hyperspectral images of brain hemodynamics, primarily to reduce the large amount of information that HSI provides. These types of statistical-based analyses are more technically familiar to applications of HSI in various fields outside biomedical applications [104].

## 3. Assessment of CMRO_2_ using HSI

2D hyperspectral measurement of brain hemodynamic response under metabolic activation (as discussed in the previous section) provides high-resolution visual information about the current oxygenation states of different cerebral regions, but fundamentally lacks the capability to quantify oxidative metabolism. Hyperspectral spatial mapping of changes in the concentrations of HbO_2_, HHb and HbT only assesses the distribution of oxygen in the brain at specific time frames, without determining the rate at which O_2_ is effectively consumed in cerebral metabolism and also the rate at which it is consequently replaced to maintain continuous fueling of neural activity. CMRO_2_ is the hemodynamic and metabolic parameter that accounts for brain oxygen consumption and its calculation requires the measurement of the CBF, to quantify the amount of O_2_ that is transported into and out of specific activated brain regions over time. A 2D mapping of CMRO_2_ thus represents a direct measure of cerebral oxidative metabolism and its variations under different circumstances, due to the targeting of molecular oxygen as an essential key element.

CMRO_2_ is generally defined as the product of CBF and the cerebral oxygen extraction factor, which is given by the net difference between cerebral arterial oxygen saturation (SaO_2_) and cerebral venous oxygen saturation (SvO_2_):

4 \begin{eqnarray*}{{\rm{CMRO}}}_{2}={\rm{CBF}}\cdot ({{\rm{SaO}}}_{2}-{{\rm{SvO}}}_{2}).\end{eqnarray*}

Both SaO_2_ and SvO_2_ depend on the measurement of other cerebral hemodynamic parameters, i.e. the concentrations of HbO_2_, HHb and HbT. Several models have been proposed over the years to express the correlation between CMRO_2_, hemodynamic parameters and CBF [105–107]. A general equation to derive the relative change ΔCMRO_2_ respect with a baseline CMRO_2_ was first established by Mayhew *et al* [107, 108]:

5 \begin{eqnarray*}\left(\displaystyle \frac{{\rm{\Delta }}{{\rm{CMRO}}}_{2}+{{\rm{CMRO}}}_{2}}{{{\rm{CMRO}}}_{2}}\right)=\left(\displaystyle \frac{{\rm{\Delta }}{[{\rm{HHb}}]}_{{\rm{v}}}+{[{\rm{HHb}}]}_{{\rm{v}}}}{{[{\rm{HHb}}]}_{{\rm{v}}}}\right)\\ \,\cdot \,\left(\displaystyle \frac{{[{\rm{HbT}}]}_{{\rm{v}}}}{{\rm{\Delta }}{[{\rm{HbT}}]}_{{\rm{v}}}+{[{\rm{HbT}}]}_{{\rm{v}}}}\right)\cdot \left(\displaystyle \frac{{\rm{\Delta }}{\rm{CBF}}+{\rm{CBF}}}{{\rm{CBF}}}\right).\end{eqnarray*}

In equation (5), [HHb]_v_ and [HbT]_v_ are the baseline concentrations of HHb and HbT in the localized brain venous compartment, while Δ[HHb]_v_ and Δ[HbT]_v_ are the corresponding time-dependent changes after metabolic activation (typically measured in *μ*M). Similarly, CBF and ΔCBF represent the baseline value of the CBF and its post-activation change (generally in mm min^–1^), respectively. Assuming that the hemoglobin concentration changes in the venous compartment are proportional to those across all the brain vasculature, a more general and simplified formula can be derived:

6 \begin{eqnarray*}\left(1+\displaystyle \frac{{\rm{\Delta }}{{\rm{CMRO}}}_{2}}{{{\rm{CMRO}}}_{2}}\right)=\left(1+{\gamma }_{r}\displaystyle \frac{{\rm{\Delta }}{[{\rm{HHb}}]}_{{\rm{v}}}}{{[{\rm{HHb}}]}_{{\rm{v}}}}\right)\\ \,\cdot \,{\left(1+{\gamma }_{t}\displaystyle \frac{{\rm{\Delta }}{[{\rm{HbT}}]}_{{\rm{v}}}}{{[{\rm{HbT}}]}_{{\rm{v}}}}\right)}^{-1}\cdot \left(1+\displaystyle \frac{{\rm{\Delta }}{\rm{CBF}}}{{\rm{CBF}}}\right),\end{eqnarray*}

where *γ*_*r*_ and *γ*_*t*_ are factors correlating the hemoglobin baselines ([HHb]_v_ and [HbT]_v_) and the hemoglobin changes (Δ[HHb]_v_ and Δ[HbT]_v_) in the venous compartments with the corresponding baselines ([HHb] and [HbT]) and changes (Δ[HHb] and Δ[HbT]) in the whole vasculature:

7 \begin{eqnarray*}{\gamma }_{r}=\left(\displaystyle \frac{{\rm{\Delta }}{[{\rm{HHb}}]}_{{\rm{v}}}}{{[{\rm{HHb}}]}_{{\rm{v}}}}\right)/\left(\displaystyle \frac{{\rm{\Delta }}[{\rm{HHb}}]}{[{\rm{HHb}}]}\right),\\ {\gamma }_{t}=\left(\displaystyle \frac{{\rm{\Delta }}{[{\rm{HbT}}]}_{{\rm{v}}}}{{[{\rm{HbT}}]}_{{\rm{v}}}}\right)/\left(\displaystyle \frac{{\rm{\Delta }}[{\rm{HbT}}]}{[{\rm{HbT}}]}\right).\end{eqnarray*}

As seen in the previous section, HSI systems are able to efficiently measure Δ[HHb] and Δ[HbT], yet they cannot provide direct quantification of CBF and its dynamic changes in response to brain metabolic demand. Therefore, combinations of HSI with other flowmetric imaging modalities capable of measuring *in vivo* CBF have been tested and investigated, to simultaneously acquire data of all the relevant quantities necessary for calculating CMRO_2_ using equation (6), thus assessing oxidative metabolism during brain activation [88, 109–112]. Laser speckle contrast imaging [113] is one of the most suitable techniques to implement in a single system together with HSI. It takes advantage of the *speckle* random interference produced by laser light after it is scattered by brain tissue. 2D detection of this scattered light produces images characterized by a granulose-looking pattern, known as *image speckle*. When the scattering of the laser light is caused by moving red blood cells, then the image speckle fluctuates between images acquired over time. Hence, the rate of variation of the speckle pattern can be mathematically correlated with the time-varying velocity of the red blood cells in each pixel, which can be used to spatially resolve and quantify CBF at specific time points [114].

**Figure 7.**
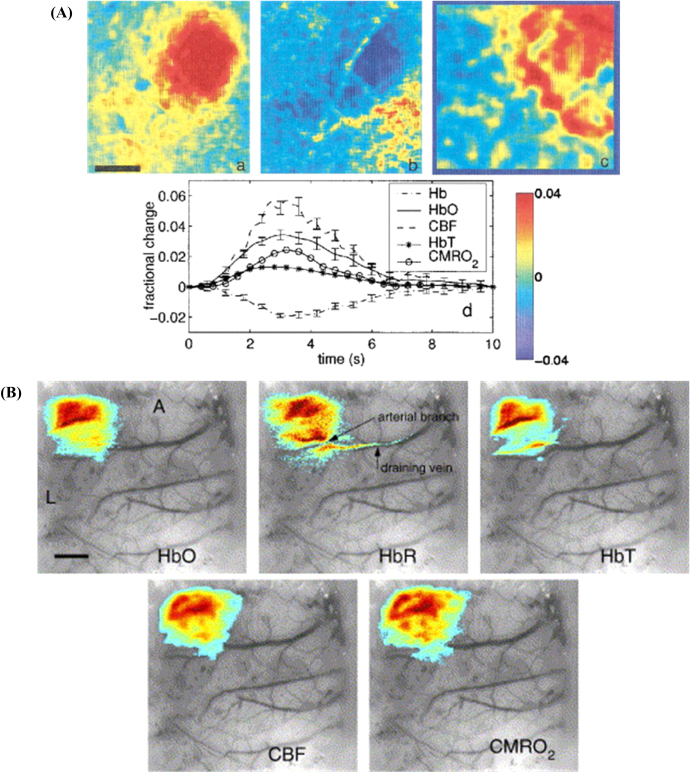
(A) Fractional change maps of HbO_2_ (a), HHb (b) and CBF (c), obtained after HSI of the exposed cortex of a mouse during single whisker stimulation. Scale bar (in black) equal to 0.5 mm. The temporal trends of the responses of HbO_2_, HHb, HbT, CBF and CMRO_2_ (d) are also shown as averaged values over the activation area. Reproduced with permission from [88], Optical Society of America. (B) Spatial extent of HbO_2_, HHb, HbT, CBF and CMRO_2_ superimposed on an image of the exposed cortex of a mouse during forepaw stimulation. The responses have been averaged around their peaking times and thresholded at 1/3 of their maximum values (A = anterior, L = lateral, scale bar = 1 mm). Reprinted from [109], Copyright (2005), with permission from Elsevier.

Dunn *et al* [88] proposed an integrated system combining laser speckle contrast imaging with the six-wavelength HSI setup that was previously described in section 2 and used to calculate metabolic-related hemodynamic changes in the exposed cortex [89]. In addition to the filtered illumination at 560, 570, 580, 590, 600 and 610 nm, they used an expanded laser diode to illuminate the exposed cortex of mice at 785 nm, in order to generate the image speckle. In earlier studies [88, 109], the speckle contrast images were acquired with the same CCD camera utilized to collect also the hyperspectral intrinsic signal from HbO_2_ and HHb. With this configuration, the imaging system was repeatedly used to image the exposed somatosensory cortex of a mouse during tactile stimulations (either forepaw or whiskers). In the first of these studies [88], only maps of the fractional changes in HbO_2_, HHb and CBF were calculated, as the ratio between the responses integrated over 3–4 s after the stimulus and the corresponding pre-stimulus baselines. As depicted in figure 7(a), the HSI-derived images localized the area of the cortex associated with the maximum rises in HbO_2_ and CBF, although the peak of the latter covers a larger, more spread and less defined region. CMRO_2_ was calculated using equation (6), assuming unitary values for *γ*_*r*_ and *γ*_*t*_. Its average temporal evolution over the activated area for all the duration of the stimulus was found to follow a similar trend to the hemodynamic response of HbO_2_. The same resulted for the time-variation of CBF, even though its response was found to be about three times greater than that of CMRO_2_.

In the second investigation with the same system [109], the spatial localization and extent of the changes in CMRO_2_ were mapped as well (figure 7(b)), and then compared with the other parameters, in order to relate metabolic and hemodynamic responses in the brain during functional activation. The peak responses of CMRO_2_, hemoglobin and CBF all showed a similar distribution in the same activated region, with CMRO_2_ covering a slightly larger area only in the preliminary phases of the stimulus onset. However, as in the previous investigation, the ratio ΔCBF/ΔCMRO_2_, indicating blood flow consumption by metabolism, was found to be equal to 1.5 and 2 for the peak responses to whiskers and forepaw stimulation, respectively. The authors suggested that this discrepancy in the responses of CBF and CMRO_2_ supported the hypothesis of an uncoupling between cerebral oxidative metabolism and brain hemodynamics during activation. Additionally, in contrast with the first study, the temporal trends of the percentage changes in CBF and CMRO_2_ showed a prolonged plateau after the peak of their respective responses.

An important question which was also targeted by the second study using the system of Dunn *et al* [109] regards the dependency of the estimated CMRO_2_ on the assumed values of *γ*_*r*_ and *γ*_*t*_. The effects of these vascular weighting constants on the spatial extent of the metabolic and hemodynamic quantities were then evaluated by varying both *γ*_*r*_ and *γ*_*t*_ from 0.5 to 2, which represents the expected physiological range of these two factors [108]. Consequently, only a small percentage change in the spatial extent of the CMRO_2_ response was estimated across the range of the *γ* factors compared to the unitary assumption (*γ*_*r*_ = *γ*_*t*_ = 1), thus implying a limited influence of the assumed vascular compartmentalization on the assessments of the cerebral oxidative metabolism.

A second version of the aforementioned system was later developed and included a secondary CCD camera for separate acquisition of the laser speckle images. This new setup was then applied to image and monitor the metabolic and hemodynamic states of the exposed cortex of a mouse during induced focal cerebral ischemia [110]. This condition resulted in much larger relative variations in both CBF and in the concentrations of HbO_2_ and HHb, compared to those associated with functional activation; thus, a first-order linear approximation of the nonlinear system derived from the MBLL (see equation (3)) was found to lead to significant errors in the calculations of the hemodynamic response. The results of the MBLL were compared with numerical simulations to account for the large increase of the optical pathlengths (more than 60%) in the ischemic core, in comparison with their corresponding baseline values. Overestimation of the decrease in the concentration of HbO_2_ during the acute ischemic-hypoxia was demonstrated with the MBLL, compared to the numerically predicted changes. Contrarily, a nonlinear fitting algorithm minimizing the difference between the measured light reflectance and that predicted through a perturbation Monte Carlo model showed better accuracy and consistency with the simulated results. Subsequently, larger drops in HbO_2_, CBF and CMRO_2_ were localized in the ischemic core right after the onset of the acute hypoxic conditions, while HHb increased as expected. A further reduction in all the hemodynamic quantities and in cerebral oxidative metabolism was also reported, spreading first to the ischemic penumbra and then to the non-ischemic areas. Persistent reduction in CMRO_2_ progressed as ischemia was maintained, even in areas unaffected by direct hypoxia. However, small residuals of metabolic activity were found to persist across the image cortex. The removal of the hypoxic conditions, allowing brain tissue reperfusion, resulted in a recovery of all the metabolic and hemodynamic parameters, although not back to pre-ischemia levels. Calculated CMRO_2_ values after reperfusion remained below those of the baseline before the hypoxic onset, thus implying a possible irreversible suppression of the metabolic activity of the affected brain tissue.

In conclusion to this section, it is worth to mention a methodology proposed by Boas *et al* [69] for indirectly estimating the variation of CMRO_2_ during metabolic activation using only measured changes in HbO_2_ and HHb derived from optical data, i.e. without the need of CBF measurements. This approach is based on the Windkessel model [115] that relates changes in CBF to changes in cerebral blood volume (CBV). From the model, it is then possible to estimate the CBF response following brain activation from optical measurements of the changes in the concentration of HbT and under the assumption that during the hemodynamic response: Δ[HbT]/[HbT] = ΔCBV/CBV. The approach was applied and validated using human fNIRS data measured at two wavelengths (682 and 830 nm) during motor stimulation. Although the methodology was shown to produced estimates of CMRO_2_ in the same range of parallel experimental findings in the literature, it also seemed to be affected by some limitations in its accuracy. In particular, the optical approach was unable to discriminate between a tight coupling of the variations of CMRO_2_ with changes in CBF and null changes in CMRO_2_ during the metabolic activation. Therefore, the authors recommended better information concerning the relationship between cerebral flow and volume in order to address these issues. Anyway, no applications of the aforementioned method to HSI have been found so far in the literature.

## 4. HSI of cerebral cellular energetics: a future perspective

An alternative approach to hyperspectral *in vivo* monitoring of brain metabolic activity and function focuses on measuring mitochondrial energetics and ATP production in the neurons directly. Instead of targeting hemodynamics parameters, such as the different states of hemoglobin and the CBF, the optical properties of different compounds involved in neuronal energy production can be exploited, in order to assess and quantify metabolism by measuring the changes in the redox states of these molecules during cellular respiration.

From the analysis of the existing literature, it has emerged that HSI of cellular metabolites is not as widespread as the previously discussed approaches. Nonetheless, it has the unique capability to provide a specific insight to the molecular processes on which the cerebral metabolic activity is based and by which it is fundamentally driven. Thus, it can be seen as a future promising direction in the application of biomedical hyperspectral monitoring to the metabolism of the living brain, particularly if coupled with the complementary information provided by the simultaneous imaging of cerebral hemodynamics and tissue oxygenation.

Fluorescent endogens such as FAD and NADH have been imaged through hyperspectral methods, yet with very few *in vivo* applications and none on the living brain. Nonetheless, HSI setups explicitly targeting fluorescence have been presented and tested, showing the capability of simultaneous imaging multiple endogenous fluorophores. This opens to the potential implementation of this methodology for cerebral metabolic monitoring.

In addition to endogenous fluorophores, HSI could also be potentially applied to target fluorescence signals from exogenous substitutes of important compounds involved in cellular energetics. Among these, one of the most promising could be 2-NBDG, a fluorescent analog of deoxyglucose with emission around 550 nm, when excited at 475 nm [116]. This compound has been used to monitor *in vivo* cerebral glucose uptake in the brain [117]. However, to our knowledge, no applications of HSI to 2-NBDG fluorescence have been found in the literature.

Similarly, the quantification of CCO *in vivo* has been largely demonstrated only through broadband spectroscopy and fNIRS techniques. The transition from broadband point-spatial approaches to 2D HSI for directly imaging the changes in the redox states of CCO is proposed here as a potential new solution for monitoring brain metabolism.

### 4.1. Fluorescence HSI of FAD and NADH

Flavoproteins (FAD/FADH_2_) and NADH represent the most targeted intrinsic biomarkers of mitochondrial metabolism in the brain cortex, due to their autofluorescence properties. The reduced form of NADH shows maximum light absorption in the range 320–380 nm, peaking at 365 nm, and it emits fluorescent light between 420 and 480 nm, with a maximum centered at 450 nm [72, 118]. Since its oxidized form, NAD (also known as NAD^+^), provides a negligible contribution to light absorption in the aforementioned range, it is possible to selectively excite NADH with UV light and detects its fluorescent emission [70]. Variations in the intensity levels of the detected fluorescence indicate changes in the concentration of the reduced state NADH associated with the rate of production of ATP in the neuronal mitochondria. A reduction in the detected fluorescent emission is typically linked to a decrease in the concentration of NADH due to its oxidation in the ETC, after cerebral metabolic activation that leads to higher energy demand by the brain. It has been demonstrated that the contribution to the fluorescence signal of mitochondrial NADH is 6–8 times greater than that of NADH in cytoplasm, thus connecting NADH fluorescence directly to the process of ATP synthesis [119]. During hypoxic conditions, NADH fluorescence and concentration increase, reaching a maximum level under complete oxygen deprivation and acute ischemia when oxidative phosphorylation is largely suppressed. On the contrary, brain hyperoxia produces a decrease in NADH due to increased oxygen supply, in the same way as during functional activation [118].

Similar to NADH, FADH_2_ is also actively involved in mitochondrial energetics as an electron donor in the ETC. However, it is its oxidized form, FAD, that exhibits fluorescence properties with emission in the range 500–560 nm, peaking at around 520 nm, while its absorption spectrum reveals two prominent bands for excitation: one between 320 and 390 nm, the other between 430 and 500 nm (the latter is the most exploited) [72, 120]. The response of FAD fluorescence emission to functional activation has been demonstrated to be biphasic [121–124]: a brief (1–2 s) increase in fluorescence intensity (light phase), corresponding to the onset of the stimulus, is generally followed by a much slower decrease in detected light (dark phase) within a few seconds after the termination of the stimulus. In case of hypoxic conditions, when metabolic activity reduces due to lack of oxygen, relative changes in the FAD fluorescence emission become evident only after significant deficiency of tissue oxygenation (FiO_2_ ≤ 10%), showing a decrease in the fluorescent signal [125].

Relative differences in the fluorescence emission of FAD and NADH compared to basal levels can then be imaged in order to localize the sites of metabolic activation and quantitatively monitor mitochondrial energy production in the brain. It has been found that the autofluorescence signal is substantially larger (10–100 times) than the intrinsic optical signal associated with hemodynamics and tissue oxygenation. The fluorescence response also shows a faster temporal course than the hemodynamic one [121, 123]. Moreover, the signal strength and the spatial resolution of the hemodynamic response typically vary across different regions of the brain according to the density of the vascular bed in each area, while the fluorescence response can be used to specifically identify metabolic changes with higher accuracy and independently of vasculature distribution [123].

It is also possible to combine the information provided by the two different fluorescence signals of FAD and NADH by simultaneous measurement: the ratio between the fluorescence intensity of the oxidized FAD and the fluorescence intensity of the reduced NADH is commonly called *fluorescence redox ratio* and provides assessment of the overall oxidation–reduction state in the mitochondria during metabolic activity. A reduction in such redox ratio commonly indicates an increase in cellular metabolic rate in response to a stimulus or during specific conditions [126, 127].

Fluorescence imaging of the living brain has been widely investigated by measuring the integrated emission intensity over relatively wide bandwidths, yet mostly without using any spectral information regarding the detected light. Nonetheless, applications of HSI to the detection of the fluorescence signals emitted by FAD and NADH can provide multiple advantages, by improving its quantitative accuracy and potentially enabling image multiplexing, i.e. the simultaneous monitoring of both the two aforementioned molecules and even of other chromophores (HbO_2_, HHb among others) relevant to metabolism [128, 129]. Furthermore, analysis of the fluorescent spectra can be used for depth discrimination and localization of the emitting sources [129].

*In vitro* and *ex vivo* studies have been performed using HSI to target fluorescent metabolites in brain cells and cerebral tissue [130–132], although *in vivo* brain functional studies have not been found in the current literature. Various proposed HSI systems have been characterized for detection of fluorescence and have shown potential fitness to target multiple endogenous fluorophores, such as FAD and NADH, with high spatial and spectral resolution performances [131–136]. For example, Gao *et al* [134] proposed a hyperspectral snapshot system for fluorescence imaging, capable of simultaneously acquiring 25 wavelength bands with 5.6 nm spectral resolution and 0.45 *μ*m spatial resolution. Snapshot acquisition was obtained through the use of an image slicer mirror assembly. The HSI system was tested on phantom models containing fluorescent beads of diameter equal to around 2.5 *μ*m and fluorescence emission peaks centered at 520 nm, corresponding to the peak of FAD.

For future *in vivo* brain applications of HSI to FAD and NADH fluorescence detection, the exposed cortex of small animals could be again the preferential target, since the near-UV and visible range is involved in both the excitation and the emission processes. However, the use of short-wavelength illumination is also the source of one of the primary issues concerning *in vivo* fluorescence imaging of NADH and FAD: in addition to the low penetration depth in the cortex, the use of UV light below 400 nm (in the case of excitation of NADH) can cause significant damage to the illuminated tissue. To overcome this problem, a combination of HSI with another optical fluorescence technique, called *two-photon fluorescence imaging*, may be advantageous [31, 137]. Two-photon fluorescence imaging is based on the use of high-output pulsed lasers emitting NIR light: when a sufficiently high photon flux is achieved, it is possible that two of the lower-energy NIR photons emitted in quick succession by the laser are absorbed simultaneously by the targeted fluorophore via a nonlinear excitation process. Consequently, the compound would emit fluorescence as if it was excited in the usual way. This technique allows NIR excitation of FAD and NADH with higher tissue penetration and eliminating the use of UV light [138]. Hyperspectral approaches to two-photon fluorescence imaging are currently being investigated [136, 139–141], opening up the possibility of moving such technology also towards *in vivo* measurements of brain metabolism.

### 4.2. HSI of cytochrome-c-oxidase

When the use of NIR light for biomedical applications was first proposed by Jöbsis [73], the primary intention was to measure *in vivo* tissue changes in the redox states of CCO, because of its fundamental role in metabolism. Following that milestone study, fNIRS technologies and methodologies have been used to specifically exploit the absorption properties of this chromophore, in order to quantify directly and non-invasively cerebral metabolic activity in both small animals and humans. Indeed, broadband NIRS has shown the capability to combine information provided by CCO with the complementary and more extensively investigated hemodynamic response (from the measurements of HbO_2_ and HHb) [60, 142]. In particular, CCO is a suitable biomarker for functional imaging of the brain, since its cerebral concentration is far higher than in other extracerebral tissues [143].

**Figure 8.**
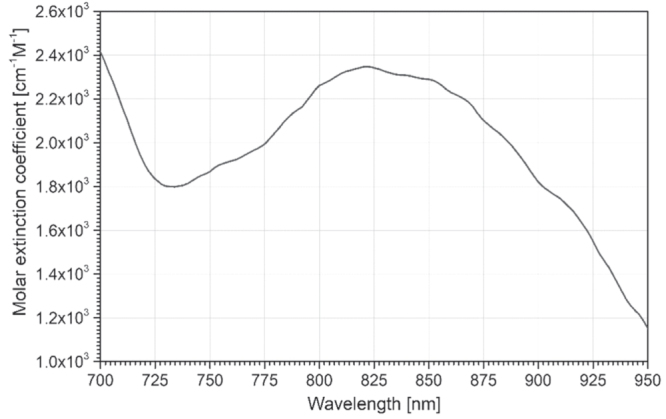
Oxidized-reduced difference molar extinction spectrum of CCO in the NIR range. The values are taken from UCL Biomedical Optics Research Laboratory database [75].

The advantage of using the NIR window to target CCO (specifically the optical signature of its copper CuA redox center between 780 and 900 nm) derives from the characteristics of the molar extinction spectra of its oxidized (oxCCO) and reduced (redCCO) forms. The total concentration of this chromophore does not vary significantly over relatively short periods of time (in the order of hours, thus much longer than the typical time trends of cerebral metabolic activation); thus, it has been demonstrated [60] that the difference absorption spectrum between oxCCO and redCCO (figure 8), combined with the measured changes in NIR light attenuation in brain tissue, can provide direct information about the relative variations in the CCO redox states *in vivo*. Particularly, concentration changes in oxCCO can be determined from the knowledge of *ε*__diffCCO__(*λ*) = *ε*__oxCCO__(*λ*) − *ε*__redCCO__(*λ*), i.e. the oxidized-reduced difference between the molar extinction coefficients of CCO (the opposite can be done for assessing changes in redCCO). As for the hemodynamic response, the same MBLL approach previously described by equation (3) in section 2 can be implemented to calculate the relative changes Δ[oxCCO] in the concentration of the oxidized species of CCO, by simply adding it as a third chromophore to the system of equations [60].

As described in section 1, the oxidation of CCO is directly correlated to production of ATP during cellular respiration. Therefore, variations in the concentration of oxCCO indicate alterations of the rate of metabolic energy production in the brain. An increase in the relative concentration of oxCCO typically corresponds to an increment in cerebral metabolism, for instance during functional activation [144–146]. Oxidation of CCO is also affected by brain oxygen supply [147]: during hypoxic conditions, oxCCO response generally presents a decrease in concentration [79, 143, 148], while an increase is typically associated with hyperoxia [149]. However, CCO is not tightly coupled with hemodynamics, due to its specificity to cellular metabolism, as well as its dependency on the availability of electrons for the ETC [147].

Several challenges are associated with the differentiation of the oxCCO signature from the hemoglobin signals, primarily due to the lower concentration of CCO in the brain (as well as in any other tissue, in general) compared to hemoglobin. Even though this is partially compensated by its higher absorption in the NIR region, the overall maximum contribution of oxCCO to the measurable optical signal is typically an order of magnitude lower than the contribution of HbO_2_ and HHb [60]. As a result of this, it is difficult to separate the signal generated by CCO redox changes from that related to the two hemoglobin species by just using few selected wavelengths. However, it has been demonstrated [150, 151] that the use of a high number of wavelengths covering the entire range of the NIR peak of the difference spectrum of CCO, from 780 to 900 nm (as seen in figure 8), significantly improves differentiation between the oxCCO and hemoglobin optical signatures, as well as overall accuracy and quality of the data.

**Figure 9.**
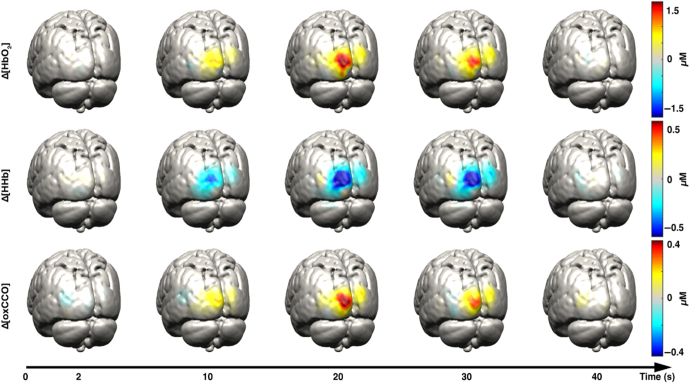
Volumetric reconstruction of spatially-resolved changes in the relative concentrations of HbO_2_ (first row), HHb (second row) and oxCCO (third row) in the left occipital cortex of human brain during visual stimulation. The temporal variations of both the hemodynamic and metabolic responses were monitored at 2, 10, 20, 30 and 40 s after the stimulus. The data were obtained using broadband fNIRS at multiple source-detector channels across the investigated brain region. Reproduced with permission from [153].

Broadband NIR spectroscopy is currently used to monitor *in vivo* brain metabolism via measurements of the concentration changes in HbO_2_, HHb and oxCCO, to achieve quantification of cerebral metabolic activation in different situations, from functional stimulation to response during oxygen-dependent conditions. Although NIRS analysis does not provide direct imaging information, recent attempts to spatially mapping brain metabolic activity have been published, using multichannel broadband spectroscopy to topographically and even volumetrically resolve changes in CCO and hemodynamics across different regions of the living cerebral cortex [152, 153]. For instance, Brigadoi *et al* [153] implemented a DOT approach to reconstruct the broadband spectroscopic data measured at fourteen different spatial locations in the left occipital cortex of human subjects during visual stimulation. The measurements were accomplished non-invasively via multiple-sources illumination of the head with white light and detection of the attenuation spectra of the diffused light. Seventeen discrete wavelengths from 740 to 900 nm at intervals of 10 nm were then chosen for the reconstruction, to limit computational burden. Tomographical maps were obtained for HbO_2_, HHb and oxCCO showing their corresponding relative changes in concentration at different time points during and after the visual stimulus. As reported in figure 9, the typical hemodynamic response to functional activation was localized in the cortex, derived from changes in HbO_2_ and HHb. The spatial and temporal variation of oxCCO was found similar to that of HbO_2_, though with lower magnitude in the concentration increase following the stimulation.

The spatially-resolved spectroscopic approach of broadband fNIRS to the measurement of oxCCO as an indicator of brain metabolism could be extended to 2D imaging and HSI has the potential to be the best candidate for this. In this perspective, HSI can be seen as the multidimensional equivalent of broadband NIRS, where every pixel of the image is associated with the corresponding spectral information. The capability of HSI to utilize multiple narrow wavelength bands in the NIR range could be used to accurately resolve the signal from oxCCO. From that, spatial distribution of the changes in the concentration of oxCCO can be monitored, thus providing a complete insight in the metabolic activity of the brain. Moreover, thanks to the NIR optical window in tissue and the smaller presence of CCO in the scalp and skull, compared to the cerebral cortex, HSI monitoring of brain metabolism could possibly be performed also non-invasively *in vivo* on humans.

**Figure 10.**
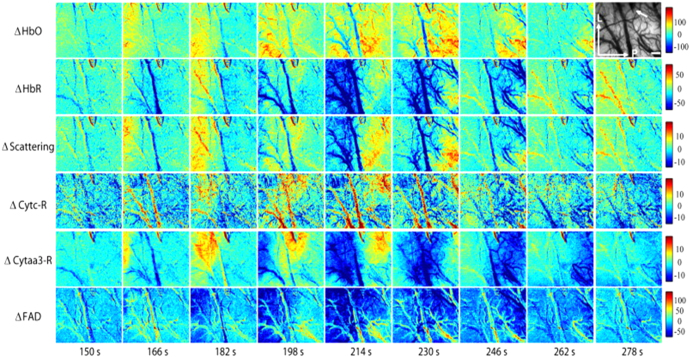
Spatial maps of the relative changes in concentration of HbO_2_ (first row), HHb (second row), scattering (third row), reduced cytochrome c (fourth row), redCCO (fifth row) and FAD (sixth row) in the exposed cortex of a rat, at different temporal intervals during and after CSD. The top-right figure represents the reflectance image at 570 nm. Scale bar (in white) equal to 0.5 mm. Reprinted from [95], Copyright (2013), with permission from Elsevier.

Currently, a single paper was found in the literature, by Yin *et al* [95], presenting a hyperspectral approach to the invasive imaging of CCO in the living brain, using visible light instead of NIR, and by targeting the spectral signature of its haem centers (collectively known as *cytochrome aa*_*3*_) instead of CuA. The article reported a very comprehensive study on the simultaneous HSI of hemodynamic response, mitochondrial metabolism and scattering changes in the exposed parietal cortex of rats during cortical spreading depression (CSD), a particular condition likely to occur in the brain following ischemia. The system used in the study was composed of a white light source and CCD camera mounted on a microscope, while LCTFs were employed to acquire images at nine different wavelengths (450, 470, 500, 530, 550, 570, 600, 630 and 650 nm). Six signals were resolved in the hyperspectral images, corresponding to the optical absorption of HbO_2_, HHb (for hemodynamics), reduced CCO (referred as *Cytaa*3-*R* in the article), FAD and reduced cytochrome c (for metabolism), plus variations in light scattering due to CSD. Cytochrome c is a chromophore that is oxidized by CCO during the ETC. Each concentration change was calculated using the MBLL with Monte Carlo estimations of the differential pathlengths. The main purpose of the study was to estimate the relative contributions of each of these signals to the overall measured optical attenuation, in order to evaluate accuracy in the quantification of the hemodynamic response during CSD. However, the study was able to provide for the first time (to our knowledge) spatial mapping of CCO redox changes in the exposed cortex. This can be seen clearly in figure 10. The absorption peaks of cytochrome aa_3_ in the visible range, at about 444, 605 and 650 nm, were targeted by the system, although such approach is more prone to noise than using NIR light, due to the predominant influence of hemoglobin in the extinction spectrum below 750 nm. Furthermore, the fluorescence emission of FAD was not accounted for. The metabolic response associated with redCCO (which has an opposite trend to oxCCO) showed an increase in its relative concentration followed by a smaller decrease before returning to baseline, corresponding to a drop in cerebral metabolism and subsequent recovery during and after CSD. The peak of the response of redCCO was also found to be almost synchronized with the hemodynamic response, as well as with the maximum variations of the other two metabolites and the scattering.

The application of HSI to metabolic monitoring of the brain via measurement of the redox states of CCO may represent a future direction in the use of this technology for imaging cerebral metabolism, especially non-invasively and with the possibility to simultaneously perform multiplexing to evaluate other significant compounds, primarily HbO_2_ and HHb.

## 5. Summary and conclusions

Past and present applications of HSI for the measurement of *in vivo* brain metabolism and hemodynamics have been reviewed: major fundamentals about the approach have been reported, particularly concerning methodologies for the quantification of the hemodynamic response and of the cerebral oxidative metabolism.

The current hyperspectral technology demonstrates superior spectral resolution performances in imaging light-absorbing chromophores, such as HbO_2_ and HHb, in the living brain, in comparison to other optical modalities. The majority of the reported studies has been primarily performed invasively, by focusing on the exposed cerebral cortex, even though this procedure has the advantage of providing much higher spatial resolution, compared to non-invasive techniques such as fNIRS and DOI. Although small animals still represent the primary targets for monitoring metabolic activity, investigations in human subjects during neurosurgery are becoming more and more common and can provide even more significant insights on brain function during a wide range of responses to stimuli and to various systemic challenges. In addition, HSI offers the important benefit of potentially targeting multiple chromophores simultaneously and perform multiplexed imaging, due to its large spectral range.

Nonetheless, drawbacks related to HSI are still present and mostly connected to instrumentation: from the complexity of the imaging equipment to computational issues related to the large volume of acquired data. Temporal resolution requirements and sensitivity to motion artifacts are also pushing the favor towards snapshot techniques for hyperspectral image acquisition.

The relatively limited amount of publications and studies on HSI for brain metabolic monitoring may be related to the recent development of the technique for medical purposes, especially concerning functional imaging and neuroscience. For a more detailed and broad review on HSI development and applications in medical sciences beyond brain imaging, we advise the reader to look at the review article by Lu *et al* [1].

Potential future solutions have also been proposed and discussed, pointing the current HSI approaches towards a more direct and specific quantification of brain metabolism that aims at monitoring *in vivo* cellular energetics and mitochondrial ATP production. Such progression in the mapping of cerebral metabolic activity could exploit the combination of the assessment of brain hemodynamics with imaging information provided by specific metabolites, such as NADH, FAD and CCO.

In conclusion, biomedical HSI is still a rapidly expanding field and new possible technological improvements could lead to novel discoveries and enhanced understanding of those mechanisms which are at the foundations of brain function.
